# The European Nucleotide Archive in 2022

**DOI:** 10.1093/nar/gkac1051

**Published:** 2022-11-18

**Authors:** Josephine Burgin, Alisha Ahamed, Carla Cummins, Rajkumar Devraj, Khadim Gueye, Dipayan Gupta, Vikas Gupta, Muhammad Haseeb, Maira Ihsan, Eugene Ivanov, Suran Jayathilaka, Vishnukumar Balavenkataraman Kadhirvelu, Manish Kumar, Ankur Lathi, Rasko Leinonen, Milena Mansurova, Jasmine McKinnon, Colman O’Cathail, Joana Paupério, Stéphane Pesant, Nadim Rahman, Gabriele Rinck, Sandeep Selvakumar, Swati Suman, Senthilnathan Vijayaraja, Zahra Waheed, Peter Woollard, David Yuan, Ahmad Zyoud, Tony Burdett, Guy Cochrane

**Affiliations:** European Molecular Biology Laboratory, European Bioinformatics Institute, Wellcome Genome Campus, Hinxton, Cambridge CB10 1SD, UK; European Molecular Biology Laboratory, European Bioinformatics Institute, Wellcome Genome Campus, Hinxton, Cambridge CB10 1SD, UK; European Molecular Biology Laboratory, European Bioinformatics Institute, Wellcome Genome Campus, Hinxton, Cambridge CB10 1SD, UK; European Molecular Biology Laboratory, European Bioinformatics Institute, Wellcome Genome Campus, Hinxton, Cambridge CB10 1SD, UK; European Molecular Biology Laboratory, European Bioinformatics Institute, Wellcome Genome Campus, Hinxton, Cambridge CB10 1SD, UK; European Molecular Biology Laboratory, European Bioinformatics Institute, Wellcome Genome Campus, Hinxton, Cambridge CB10 1SD, UK; European Molecular Biology Laboratory, European Bioinformatics Institute, Wellcome Genome Campus, Hinxton, Cambridge CB10 1SD, UK; European Molecular Biology Laboratory, European Bioinformatics Institute, Wellcome Genome Campus, Hinxton, Cambridge CB10 1SD, UK; European Molecular Biology Laboratory, European Bioinformatics Institute, Wellcome Genome Campus, Hinxton, Cambridge CB10 1SD, UK; European Molecular Biology Laboratory, European Bioinformatics Institute, Wellcome Genome Campus, Hinxton, Cambridge CB10 1SD, UK; European Molecular Biology Laboratory, European Bioinformatics Institute, Wellcome Genome Campus, Hinxton, Cambridge CB10 1SD, UK; European Molecular Biology Laboratory, European Bioinformatics Institute, Wellcome Genome Campus, Hinxton, Cambridge CB10 1SD, UK; European Molecular Biology Laboratory, European Bioinformatics Institute, Wellcome Genome Campus, Hinxton, Cambridge CB10 1SD, UK; European Molecular Biology Laboratory, European Bioinformatics Institute, Wellcome Genome Campus, Hinxton, Cambridge CB10 1SD, UK; European Molecular Biology Laboratory, European Bioinformatics Institute, Wellcome Genome Campus, Hinxton, Cambridge CB10 1SD, UK; European Molecular Biology Laboratory, European Bioinformatics Institute, Wellcome Genome Campus, Hinxton, Cambridge CB10 1SD, UK; European Molecular Biology Laboratory, European Bioinformatics Institute, Wellcome Genome Campus, Hinxton, Cambridge CB10 1SD, UK; European Molecular Biology Laboratory, European Bioinformatics Institute, Wellcome Genome Campus, Hinxton, Cambridge CB10 1SD, UK; European Molecular Biology Laboratory, European Bioinformatics Institute, Wellcome Genome Campus, Hinxton, Cambridge CB10 1SD, UK; European Molecular Biology Laboratory, European Bioinformatics Institute, Wellcome Genome Campus, Hinxton, Cambridge CB10 1SD, UK; European Molecular Biology Laboratory, European Bioinformatics Institute, Wellcome Genome Campus, Hinxton, Cambridge CB10 1SD, UK; European Molecular Biology Laboratory, European Bioinformatics Institute, Wellcome Genome Campus, Hinxton, Cambridge CB10 1SD, UK; European Molecular Biology Laboratory, European Bioinformatics Institute, Wellcome Genome Campus, Hinxton, Cambridge CB10 1SD, UK; European Molecular Biology Laboratory, European Bioinformatics Institute, Wellcome Genome Campus, Hinxton, Cambridge CB10 1SD, UK; European Molecular Biology Laboratory, European Bioinformatics Institute, Wellcome Genome Campus, Hinxton, Cambridge CB10 1SD, UK; European Molecular Biology Laboratory, European Bioinformatics Institute, Wellcome Genome Campus, Hinxton, Cambridge CB10 1SD, UK; European Molecular Biology Laboratory, European Bioinformatics Institute, Wellcome Genome Campus, Hinxton, Cambridge CB10 1SD, UK; European Molecular Biology Laboratory, European Bioinformatics Institute, Wellcome Genome Campus, Hinxton, Cambridge CB10 1SD, UK; European Molecular Biology Laboratory, European Bioinformatics Institute, Wellcome Genome Campus, Hinxton, Cambridge CB10 1SD, UK; European Molecular Biology Laboratory, European Bioinformatics Institute, Wellcome Genome Campus, Hinxton, Cambridge CB10 1SD, UK; European Molecular Biology Laboratory, European Bioinformatics Institute, Wellcome Genome Campus, Hinxton, Cambridge CB10 1SD, UK

## Abstract

The European Nucleotide Archive (ENA; https://www.ebi.ac.uk/ena), maintained by the European Molecular Biology Laboratory's European Bioinformatics Institute (EMBL-EBI), offers those producing data an open and supported platform for the management, archiving, publication, and dissemination of data; and to the scientific community as a whole, it offers a globally comprehensive data set through a host of data discovery and retrieval tools. Here, we describe recent updates to the ENA’s submission and retrieval services as well as focused efforts to improve connectivity, reusability, and interoperability of ENA data and metadata.

## INTRODUCTION

The European Nucleotide Archive (ENA; https://www.ebi.ac.uk/ena) has been established for 40 years as a database of record for nucleotide sequence and related information. It is a freely accessible and global platform for managing nucleotide sequence data and offers a comprehensive set of data submission, discovery and dissemination services.

The ENA is a founder and a partner in the International Nucleotide Sequence Data Collaboration (INSDC) (1), a long-standing data exchange initiative with partners in the National Center for Biotechnology (NCBI) (2) in the United States and the DNA DataBank of Japan (DDBJ) (3). Through our activities with the INSDC, we continue to engage with the scientific community and our user-base in developing and implementing best practices for sequence archiving.

ENA content is global, representing the interests of scientists in all parts of the world with over 92% of the world's countries represented in the user-base.

In 2022, we have continued to develop new data types as the world of sequencing and bioinformatics progresses; focused on improving data linking to further data connectivity and traceability; and endeavoured to build better recognition for users’ data sharing efforts.

## ENA CONTENT AND SERVICES

The ENA provides services for the submission and retrieval of sequence data including but not limited to: the Webin submissions portal, the Webin command line client and the Webin REST API; the ENA browser and web services for data display, search and discovery; the support helpdesk with associated training and user documentation. Table 1 summarises the entry points for ENA’s key services.

**Table 1.** Description of ENA services and their entry points

Service	Entry points	Purpose	Links
User Support	Support web form	Helpdesk services: contact and feedback	https://www.ebi.ac.uk/ena/browser/support
	Support documentation	Comprehensive documentation on data submission, update and retrieval and other FAQs	https://www.ebi.ac.uk/ena/browser/guides
Data submission	Submission tools	Submission guide and submission management tools	https://www.ebi.ac.uk/ena/browser/submit
Data access	ENA Browser	Tools to discover, search, filter and access data of many types	https://www.ebi.ac.uk/ena/browser/search

In the past 12 months, the ENA has supported 2721 submission accounts, both individual submitters and large-scale brokers, from over 80 different countries. This spans over 11 million individual submissions and 8036 studies, including over 4.1 million samples, 2.6 million runs and 3.1 million assemblies with an average of 31 unique submitters a day and an average of 24 129 daily submissions.

Data are submitted to the ENA using the Webin submission service introduced >20 years ago. Webin supports both small-scale and high-throughput submitters, and includes the Webin Portal for interactive submissions (https://www.ebi.ac.uk/ena/submit/webin), Webin REST for programmatic submissions (https://www.ebi.ac.uk/ena/submit/drop-box), Webin-CLI for command-line submissions (https://github.com/enasequence/webin-cli/releases), and also Webin-CLI REST currently for SARS-CoV-2 programmatic submissions (https://www.ebi.ac.uk/ena/submit/webin-cli). For programmatic submitters, Webin also provides reporting (https://www.ebi.ac.uk/ena/submit/report) and authentication (https://www.ebi.ac.uk/ena/submit/webin/auth) services.

In the last year, we have introduced a new version of the Webin REST programmatic submission service (https://www.ebi.ac.uk/ena/submit/webin-v2) which provides both synchronous and asynchronous submission endpoints. The asynchronous endpoint supports larger submissions and better load balancing in case of high submission loads. This new API also supports the submission of multiple XMLs of different data types together (for example, a combination of studies, samples, experiments and runs) in the HTTP POST request body, rather than only a single object type using HTTP POST multipart/form-data. This can assist users to streamline their submissions of a whole study and its components into a single submission. In the future, we also plan to introduce JSON support alongside the existing XML support. We encourage programmatic submitters to transition to these new endpoints. Further information about the new Webin REST service is available from: https://ena-docs.readthedocs.io/en/latest/submit/general-guide/programmatic-v2.html.

For presentation and data access, the ENA Browser is used by over 40,000 monthly visitors and an average of 349 619 GB of data is downloaded each month. Our presentation services include widely used RESTful APIs for searching and retrieving data. During the past year we have made improvements to our pathogen data presentation, file downloading, and the ENA Advanced Search and cross-reference services.

We have released a new and improved version of our Pathogen Portal (https://www.ebi.ac.uk/ena/pathogens/v2). This aims to bring together pathogenic data resources not only from the ENA but also from other EMBL-EBI resources, and provide rapid access to available data for new outbreaks. The new Pathogen Portal also includes the Infectious Diseases Cohort Browser (https://www.ebi.ac.uk/ena/pathogens/v2/cohorts), supported by ReCODiD (https://recodid.eu/).

We have made several improvements to the ena-file-downloader command line tool including support for downloading private data from ENA data hubs to share pre-publication data between registered users in pathogen data hubs (https://github.com/enasequence/ena-ftp-downloader/).

We have expanded the ENA Advanced Search API to include several new metadata fields, including the retrieval of primary publications information from sequence flat files (https://www.ebi.ac.uk/ena/browser/advanced-search?result=sequence&query=pubmed_id%3D%22*%22&fields=pubmed_url,pubmed_id). We have also improved access to genome assemblies by providing a single route to retrieve all sequences of a genome assembly in one go, both through the browser and programmatically via the API (e.g. https://www.ebi.ac.uk/ena/browser/api/embl/GCA_000001215.4?download=true&gzip=true).

Other improvements include adding new links to the ENA cross-reference service between ENA data and BacDive Metadatabase as well as Gene Expression Omnibus (https://www.ebi.ac.uk/ena/browser/xref).

At the time of writing, the number of raw data records within the ENA exceeds 22 million and ENA contains >2.7 billion assembled/annotated sequences. In 2021, ENA accessions were cited in 24 232 publications and the volume of archived data exceeds 40 Petabytes.

## SELECTED DEVELOPMENTS IN 2022

The European Nucleotide Archive is a voice of open data advocacy and recognises the growing importance for data generators to receive recognition for their data contributions as well as recognition of the origin of the source of sequence data within the open public archives.

In late 2021, INSDC announced an aim to put in place additional requirements to increase the submission of accurate geographical annotation and collection date information and also to harmonise these data in our records. Our ultimate goal is to ensure spatio-temporal annotations are collected for all new incoming samples and sequences, and so to significantly increase the number of records where the origin is annotated. The ENA has also been making strides towards better validation for metadata links to source material in culture collections and biobanks to produce clearer links to the biological source material of records.

In addition to our goals to improve reusability and traceability of the data, we have also improved the interoperability of our data both with other EMBL-EBI services: progressing in our goal to centralise EMBL-EBI BioSamples, as well as further afield through expansion of our global network of brokering facilities to facilitate data management and submission.

The ENA has also begun taking the first steps in our long-term aim to introduce accepting GFF3 submissions, to further increase interoperability with annotation services such as Ensembl, and also simplify the submission of genomes and annotation.

### Building recognition and credit for data sharing

To improve the visibility of the contributions researchers and data providers make to open data, the ENA has developed features that allow its users to get recognition for datasets. The ORCID (https://orcid.org/) system will be familiar to many as a method for claiming publications, grants and other types of contributions against an ID unique to each individual. ENA studies (projects) can now be claimed against a user's ORCID ID, in the same way that publications can. This is implemented through the EBI Search search interface: https://www.ebi.ac.uk/ebisearch/orcidclaimdocumentation.ebi.

The ENA is well established as an open access repository for sequence data. We continue to request that all authors refer to ENA records in publications using accession numbers issued directly by the ENA (see https://www.ebi.ac.uk/ena/browser/about/citing-ena), in order to provide recognition and credit for the open sharing of sequence data. Additionally, the ENA has brought in support for generating Digital Object Identifiers (DOIs) specifically for SARS-CoV-2 projects. In these circumstances, a DOI is issued for ENA records through an associated BioStudies record created on behalf of the submitter, that collects all relevant ENA projects (https://www.ebi.ac.uk/biostudies/about). However, DOIs are not routinely issued for ENA or BioStudies records, so to request a DOI for a SARS-CoV-2 dataset, users must contact the SARS-CoV-2 helpdesk through virus-dataflow@ebi.ac.uk.

### Improving references to source material

The ENA encourages the submission of metadata referring to the biological source of sequence data through sample attributes and sequence source feature qualifiers (https://www.insdc.org/submitting-standards/feature-table/). However, this metadata is not always complete, or may be ambiguous, preventing the direct linking of sequence data to their biological origin. We have developed a publicly accessible open-source tool, the ENA Source Attribute Helper API, to help users validate and construct these source attributes (https://www.ebi.ac.uk/ena/sah/api/) (4). This first version of the API focuses on the source attributes that identify specimens, culture collections or other biological material from which the sequence data was derived, namely on the /specimen_voucher, /culture_collection and /bio_material attributes. This tool was developed within the scope of the BiCIKL project (Biodiversity Community Integrated Knowledge Library) (5), a Horizon 2020 project that aims towards an interlinked body of FAIR (Findable, Accessible, Interoperable and Reusable) data for biodiversity research, covering different data resources e.g. molecular biology, natural history collections, taxonomy, and literature. The Source Attribute Helper API is expected to contribute to the enrichment of ENA metadata increasing overall reusability and discoverability of sequence data.

There are plans to integrate this API into submission services with development of a User Interface underway at the time of writing.

### Continued integration of BioSamples

In the last year, the ENA has continued our ongoing work to normalise sample data management into the central EMBL-EBI service, BioSamples (6).

As part of this, EBI BioSamples now supports Webin authentication. This allows submitters to register samples in EBI BioSamples using their Webin credentials and to then use these samples in ENA submissions while they remain in pre-publication private state. Once data in the ENA becomes public, the associated BioSamples will become automatically public. In our presentation services, we now display the BioSamples record in the ENA browser and the BioSamples accession (SAME- format accession) is considered the primary accession. These improvements are part of our roadmap to deepen the integration between EBI BioSamples and move away from samples registered specifically in the ENA, which will allow the use of common BioSample records in cross-omics studies.

As well as the above technical changes, the ENA has adopted the BioSamples standard model for defining relationships between samples to model ‘derived from’ hierarchies of samples and ‘composed of’ groupings of samples. Although it was previously possible to include these relationships in the ENA sample metadata when provided, this now propagates a ‘relationship’ link within the BioSamples service allowing easier navigation between related samples. Further to this, within the BioSamples service, when a relationship exists, metadata are automatically copied down from ‘parent’ to ‘child’ providing display of common metadata. There are plans to also integrate this copy-down display into the ENA Browser in future, which will further simplify use of this relationship model going forwards.

This model has already been used extensively within metagenome assemblies to provide links back from a submitted Metagenome-Assembled Genome or a binned assembly, to its source environmental sample. The availability of the sample in BioSamples will make these links more accessible. The model has also been used to link tissue-level samples to specimens in biodiversity initiatives, for example, so tissue-specific transcriptomic RNA-seq data can be linked to a specimen-level genome assembly. More recently, in light of the COVID-19 pandemic, it is being used to create links between pathogen data and associated patient data in restricted access archives such as the European Genome-Phenome Archive (EGA) (7). For pathogen data, BioSamples can now be ‘derived from’ a proxy intermediate BioSample that represents a minimal representation of the patient (e.g. https://www.ebi.ac.uk/biosamples/samples/SAMEA12928721), which will then be directly linked to the restricted access metadata and data. This provides better connectivity of the data in the two archives and enables users to trace a pathogen record back to the origin material.

### Expansion of the ENA brokering network

The ENA has expanded its brokering network over recent years to a total of 60 brokers, consisting of sequencing facilities, national infrastructure and research institutes. Brokers are specialist ENA users who can submit data and metadata to the ENA on behalf of other people and institutes. Broker facilities drive good metadata compliance and can provide local or domain specific data and metadata management, which in turn enriches, standardises, and further validates the metadata and data in public archives.

During the COVID-19 pandemic, we found that in many cases a central national brokering facility provided the most efficient means of data management at the required scale. This is particularly exemplified through the success of our first national broker of SARS-CoV-2 data, the COVID-19 Genomics UK Consortium (COG-UK), who since early 2020 have brokered the highest volume of COVID data to date (2 600 430 raw reads and 2 176 970 assembled sequences).

Since then, this has grown to seven national brokers who have set up long-term SARS-CoV-2 data streams to the ENA, including the Robert Koch Institute (RKI) in Germany, the Irish Consortium for Sequencing Covid, and the Swiss Institute for Bioinformatics (SIB). Many of these organisations liaise with their national public health institutes as part of their setup. In terms of submissions, RKI are the second biggest contributors of assembled sequences to the ENA, totalling 503 645 sequences, while Irish and Swiss groups brokered the second highest volume of raw reads after COG-UK, totalling 76 095 and 57 827 reads, respectively.

Some brokers also utilise some of our dedicated SARS-CoV-2 submission tools, such as the Webin-CLI Rest API (https://www.ebi.ac.uk/ena/submit/webin-cli) to facilitate high-volume assembly data sharing, and the GISAID to ENA sample conversion script (https://github.com/enasequence/ena-content-dataflow/tree/master/scripts/gisaid_to_ena) together with the advised ‘GISAID Accession ID’ sample attribute, to enhance the access of data already submitted to GISAID.

We have also seen success through our biodiversity collaborations. For example, the COPO service (8) developed at the Earlham Institute in the UK provides a centralised service for sample metadata management including for many biodiversity monitoring projects such as the Darwin Tree of Life (DToL) (9) and the Aquatic Symbiosis Genomics (ASG) (10) projects. Both these projects aim to generate sequence data and reference genomes for a large set of organisms and have required a large collaborative effort to collect and coordinate samples from different locations, collected and identified by many different people. COPO has been a broker since 2014 and has provided these initiatives a place to collate this information and to manage metadata and taxonomic assignments for samples providing further standardisation and validation of the metadata.

Expansion of the ENA’s brokering network distributes the responsibility for data management, making submission to the ENA more accessible, and we hope to see continued evolution of this network.

### Adding support for decoupled annotation

The ENA has long-accepted genome assembly sequences and annotations as single flat file records in the INSDC-defined feature table format (https://www.insdc.org/submitting-standards/feature-table/). Submission and update of genome assemblies to the ENA currently requires representation of both sequence and annotation with each new version. To make the submission and updating of genome annotation more accessible to users, the ENA is developing a new system to decouple the annotation from the sequence. As part of this, we recognise that many modern annotation tools output tab-delimited formats such as GFF3, and plan to support submission of annotation to the ENA in the GFF3 format. A submittable GFF3 format specification is being developed in collaboration with the Ensembl (11) service at EMBL-EBI and our partners at INSDC to ensure that the design of new annotation structure aligns with Ensembl annotation standards and also to ensure that the ENA-submitted GFF3 annotation will be shareable across INSDC partners.

This new model will enable annotations submitted through the ENA to be added to an existing assembly in INSDC, opening the avenue for users to assert different perspectives and different types of annotation on the same assembly. It would also enable independent versioning for sequence and annotation records allowing easier referencing in publication and tracking of changes.

In its initial design, decoupled GFF3 annotation will be deployed as a pilot data type used to distribute and share Ensembl's annotations generated as part of the Darwin Tree of Life project. DToL aims to sequence the genomes of 70 000 eukaryotic species in Britain and Ireland, and the project will enable the ENA to test the scalability and ease-of-use of this new system with the aim to roll this out for wider users in the future.

## DATA AVAILABILITY

ENA services are freely available at (http://www.ebi.ac.uk/ena). Content is distributed under the EMBL-EBI Terms of Use available at (https://www.ebi.ac.uk/about/terms-of-use).

## Notes

Present address: Josephine Burgin, European Molecular Biology Laboratory, European Bioinformatics Institute, Wellcome Genome Campus, Hinxton, Cambridge CB10 1SD, UK.
